# Interferon regulatory factor 1 enhances T cell differentiation in patients with myasthenia gravis

**DOI:** 10.4103/NRR.NRR-D-24-01646

**Published:** 2025-10-30

**Authors:** Yuebei Luo, Yijun Ren, Zeyi Wen, Zhaohui Luo, Huan Yang, Liqun Xu

**Affiliations:** 1 Department of Neurology, Xiangya Hospital, Central South University, Changsha, Hunan Province, China; 2 Department of Neurology, Xiangya Hospital, Central South University, Jiangxi (National Regional Center for Neurological Diseases), Nanchang, Jiangxi Province, China; 3 National Clinical Research Center for Geriatric Disorders, Xiangya Hospital, Central South University, Changsha, Hunan Province, China; 4 Research Center for Neuroimmune and Neuromuscular Disorders, Xiangya Hospital, Central South University, Changsha, Hunan Province, China

**Keywords:** autoimmune condition, B cell, CD180, histone deacetylase 1, interferon regulatory factor 1, mitogen-activated protein kinase, myasthenia gravis, nuclear factor-kappa B, T cell, Toll-like receptor 4

## Abstract

Interferon regulatory factor 1 is involved in many autoimmune conditions and is increased in patients with myasthenia gravis. However, its function in myasthenia gravis remains unclear. Herein, we explored the function of interferon regulatory factor 1 in myasthenia gravis, with an aim to understand the underlying mechanisms. Patients with myasthenia gravis who had acetylcholine receptor antibodies were included in the study. Peripheral blood lymphocytes were extracted from the included patients, and B lymphocyte subsets were isolated. Next, T and B cells from peripheral blood were co-cultured to explore the interferon regulatory factor 1-related mechanisms in myasthenia gravis. Chromatin immunoprecipitation experiments confirmed an interaction between interferon regulatory factor 1 and the *CD180* promoter region. Dual-luciferase reporter gene confirmed the transcriptional activity of interferon regulatory factor 1 on *CD180* promoter. *In vitro* results further indicated that interferon regulatory factor 1 promoted B cell activation and T cell differentiation via the inhibition of CD180. Interferon regulatory factor 1 recruited histone deacetylase 1 to inhibit CD180 transcription. Additionally, histone deacetylase 1 promoted B cell activation and T cell differentiation. Finally, *in vitro* experiments demonstrated that CD180 inhibited B cell activation and T cell differentiation by inhibiting the Toll-like receptor 4/mitogen-activated protein kinases/nuclear factor-kappa B pathway. Collectively, our results suggest that interferon regulatory factor 1 enhances T cell differentiation by recruiting histone deacetylase 1 to block B cell *CD180* transcription in myasthenia gravis via the Toll-like receptor 4/mitogen-activated protein kinases/nuclear factor-kappa B pathway. Together, these findings indicate the important role of interferon regulatory factor 1 in myasthenia gravis and suggest its molecular mechanisms. They also provide new ideas and targets for diagnosing and treating myasthenia gravis, which will be both scientifically and clinically valuable.

## Graphical abstract

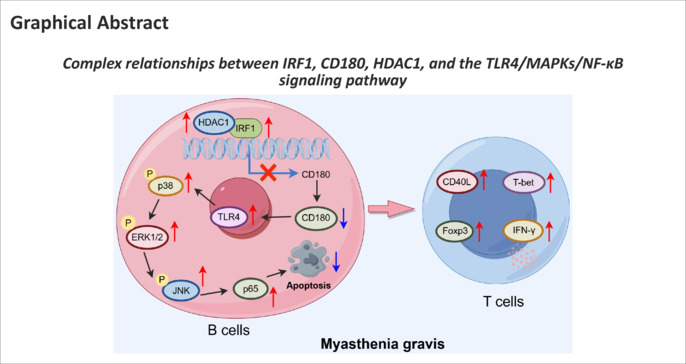

## Introduction

Myasthenia gravis (MG) is a somatically acquired autoimmune disorder that is defined by the generation of autoantibodies that impair neuromuscular transmission at the motor endplate (Zhu et al., 2023). Its clinical features include fatigue and weakness of the ocular, axial, limb, and/or respiratory muscles (Vanoli and Mantegazza, 2023). MG can affect both physical and mental functioning as well as health-related quality of life (Dewilde et al., 2023). Various factors may contribute to the development of MG, such as infections, immune reactions, surgery, and medications (Beloor Suresh and Asuncion, 2025). The thymus is crucial for the development of the disease, and most of the commonly detected pathogenic autoantibodies are against the acetylcholine receptor (AChR) (García Estévez and Pardo Fernández, 2023). MG treatment is usually personalized based on disease severity, antibody status, comorbidities, and other factors (Morren and Li, 2023). However, despite research efforts, the precise mechanisms underlying MG remain incompletely understood. Furthermore, there are risks associated with comprehensive immunosuppression, and a subset of patients with MG are refractory to current treatments.

The immunological mechanisms underlying MG development are diverse. They involve both genetic predisposition and acquired immune dysregulation, including abnormalities in cytokine secretion, T helper 1/2 imbalance, and impaired regulatory T lymphocyte function (Weiss et al., 2016). Stimulated T cells, B cells, and plasma cells, along with cytokines, contribute substantially to the generation of disease-causing autoantibodies as well as the onset of inflammation at the neuromuscular junction in MG (Uzawa et al., 2021). CD180 was initially identified on human B cells through its interaction with a mouse monoclonal antibody (clone G28.8) (Valentine et al., 1988). Also known as RP105, CD180 is a member of the Toll-like receptor (TLR) family and is mainly localized on mature B cells, monocytes, and specific dendritic cells, which are antigen-presenting cells. It is essential for innate immunity, as it detects the invariant structures of pathogens and initiates subsequent signaling pathways (Ogata et al., 2000). Abnormal CD180 expression is associated with the overactivation of B and T cells, which may lead to exacerbated autoimmune responses (Edwards et al., 2023). A previous study has reported that CD180^–^ B cells are highly activated and can induce autoantibody production in individuals with autoimmune conditions such as systemic lupus erythematosus, whereas CD180^+^ B cells cannot. This finding suggests that CD180^–^ B cells may be associated with autoantibody and immunoglobulin (Ig) production (Yang et al., 2018). However, there has been limited research into the association between CD180 B cell subsets and the development and treatment of MG. Elucidating the regulatory mechanisms of pathogenic antibody-producing B lymphocytes in the development of MG may therefore offer vital insights that allow for the development of targeted therapeutic approaches for MG.

The family of interferon regulatory factors (IRFs) is an important group of transcriptional regulators that are responsible for the production of type I interferons (IFNs) and the modulation of both innate and adaptive immune reactions (Xia et al., 2020). These factors are crucial for triggering innate immune responses to pathogens and for shaping subsequent adaptive immune reactions. Abnormalities in IRF signaling have been associated with the development of autoimmune conditions (Matta et al., 2017). IRFs constitute a family of transcriptional regulators that possess a distinctive, unconventional helix-turn-helix DNA-binding domain (Taniguchi et al., 2001). The IRF family of transcription factors control the entire IFN system, from the induction of IFNs to a variety of IFN responses, thus providing the primary basis for host resistance to pathogens (Ozato et al., 2007).

IRF1 is a component of the IRF family; it was originally discovered as a nuclear factor that interacts with and enhances the activity of *IFN* gene promoters (Feng et al., 2021). IRF1 is reported to regulate various physiological responses in cellular defense processes, including immune responses to viral and bacterial infections, the regulation of inducible gene expression, lymphocyte development and function, cell cycle regulation, and the induction of growth inhibition and apoptosis (Ohmori et al., 1997). For example, IRF1 plays a crucial role in regulating the activation and differentiation of T and B cells and the production of cytokines (Giang and La Cava, 2017). Recent studies have also revealed that IRF1 exerts a crucial effect in autoimmune diseases (such as systemic lupus erythematosus and rheumatoid arthritis) by modulating signaling pathways in immune cells (Leung et al., 2020; Wang et al., 2024). Although it is clear that IRF1 influences the growth and maturation of lymphocytes and is important in multiple autoimmune diseases, its specific function in B lymphocytes within the context of MG has not yet been reported. Using the JASPAR website (https://jaspar.elixir.no/; Rauluseviciute et al., 2024), we predicted a potential interaction between *IRF1* and *CD180* at the structural level, with *IRF1* identified as a transcription factor binding upstream of *CD180* (unpublished data). Furthermore, we speculate that IRF1 may modulate CD180 expression to influence the distribution of CD180^–^B and CD180^+^ B cells and antibody secretion, thereby participating in the development of MG. Elucidating the IRF1/CD180 regulatory axis in the context of MG pathogenesis might therefore have important implications for understanding the disease.

TLRs are instrumental for triggering innate immune responses by identifying distinctive patterns present in microbial molecules (Takeda and Akira, 2004). TLR4 stimulation can reportedly provoke an innate immune reaction and trigger the nuclear factor-kappa B (NF-κB) signaling cascade, resulting in the secretion of numerous pro-inflammatory cytokines and widespread inflammation (Lawrence, 2009). In addition, CD180 can mitigate damage from hypoxia/reoxygenation in cardiac microvascular endothelial cells by repressing the TLR4/mitogen-activated protein kinases (MAPKs)/NF-κB axis (Guo et al., 2018). We therefore further speculate that IRF1 may modulate CD180 expression and affect the distribution of CD180^−^ B and CD180^+^ B cells, as well as antibody secretion and other functions, through its modulation of the TLR4/MAPKs/NF-κB signaling pathway, which is involved in the occurrence and development of MG.

Based on the aforementioned background, the aim of the present research was to investigate whether IRF1 increases the number of CD180^–^ B cells by downregulating CD180, thereby mediating T cell activation and exacerbating MG through the TLR4/MAPKs/NF-κB signaling pathway. Our investigation may establish a foundation for the development of targeted MG treatments and presents novel avenues for exploring the etiology of the disease and clinical intervention strategies.

## Methods

### Clinical sample collection

In the present study, we recruited patients with MG who tested positive for AChR antibody in Xiangya Hospital, Central South University, from April 2024 to July 2024. The inclusion criteria for participation were as follows: aged 18–65 years, positive for MG anti-AChR antibodies, positive for serum AChR antibodies, positive neostigmine test, typical clinical symptoms, non-thymoma patients scheduled for thymectomy, and consented to be part of the research. The exclusion criteria for participants were as follows: pregnant or lactating women, or those intending to conceive shortly; patients with severe cardiopulmonary, liver, or kidney diseases, acute or chronic infections, and autoimmune diseases (such as hyperthyroidism); patients with mental disorders whom the clinical physician deemed unsuitable for participation in this study; and patients who declined to take part in the research. The study protocol was approved by the Medical Ethics Committee of Xiangya Hospital, Central South University (approval No. 2022020478). The research was conducted according to the World Medical Association *Declaration of Helsinki*. All participants provided informed consent before sampling.

### Isolation and stimulation of B cells and CD4^+^ T cells in human peripheral blood mononuclear cells

Heparinized venous blood from patients with MG undergoing lymphoplasmic replacement therapy was obtained, and peripheral blood mononuclear cells were freshly isolated using Ficol-Histopaque (Sigma-Aldrich, St. Louis, MO, USA) density gradient centrifugation. B cells and CD4^+^ T cells were obtained using magnetic bead sorting (Miltenyi Biotec, Bergisch Gladbach, Germany). Subsets of CD180^–^ B cells and CD180^+^ B cells were purified using flow cytometric sorting, following the manufacturer’s guidelines (Miltenyi Biotec). CD4^+^ T cells were then cultured in RPMI-1640 medium (R6504, Sigma-Aldrich) with 10% fetal bovine serum. These cells were activated with phorbol 12-myristate 13-acetate (PMA, 2 µg/mL, P1585, Sigma-Aldrich) and ionomycin (5 µg/mL, I0634, Sigma-Aldrich) for 4 hours.

### Cell culture and treatment

First, RNA interference of IRF1, CD180, and histone deacetylase 1 (HDAC1) and overexpression of IRF1, CD180, and HDAC1 were performed. The transfection process was conducted using Lipofectamine 2000 (11668019, Invitrogen, Carlsbad, CA, USA) following the manufacturer’s protocol.

CD4^+^ T cells were activated with PMA (2 µg/mL) and ionomycin (5 µg/mL) for 4 hours. Subsequently, CD180^–^ B lymphocytes were subjected to transfection with short hairpin negative control (sh-NC)/sh-*IRF1*, and/or sh-NC/sh-*CD180*, whereas CD180^+^ B lymphocytes were treated with overexpression (oe)-NC/oe-*IRF1* and/or oe-NC/oe-*CD180*, followed by the co-culturing of B and T cells. The interference experimental setup was as follows: Control group (CD180^–^ B lymphocytes cultured under standard conditions), sh-NC group (CD180^–^ B lymphocytes transfected with sh-NC), sh-*IRF1* group (CD180^–^ B lymphocytes transfected with sh-*IRF1*), sh-*IRF1* + sh-NC group (CD180^–^ B lymphocytes co-transfected with sh-*IRF1* and sh-NC), and sh-*IRF1* + sh-*CD180* group (CD180^–^ B lymphocytes co-transfected with sh-*IRF1* and sh-*CD180*). The overexpression experimental setup was as follows: Control group (CD180^+^ B lymphocytes cultured under standard conditions), oe-NC group (CD180^+^ B lymphocytes transfected with oe-NC), oe-*IRF1* group (CD180^+^ B lymphocytes transfected with oe-*IRF1*), oe-*IRF1* + oe-NC group (CD180^+^ B lymphocytes co-transfected with oe-*IRF1* and oe-NC), and oe-*IRF1* + oe-*CD180* group (CD180^+^ B lymphocytes co-transfected with oe-*IRF1* and oe-*CD180*).

CD4^+^ T cells were stimulated with PMA (2 µg/mL) and ionomycin (5 µg/mL) for 4 hours. Following this, CD180^–^ B lymphocytes underwent transfection with sh-NC/sh-*IRF1* and/or oe-NC/oe-*HDAC1*, whereas CD180^+^ B lymphocytes were transfected with oe-NC/oe-*IRF1* and/or sh-NC/sh-*HDAC1* prior to being co-cultured with T cells. The experimental groups were as follows: sh-NC group (CD180^–^ B lymphocytes transfected with sh-NC), *sh-IRF1* group (CD180^–^ B lymphocytes transfected with sh-*IRF1*), sh-NC + oe-*HDAC1* group (CD180^–^ B lymphocytes transfected with sh-NC and oe-*HDAC1*), and sh-*IRF1* + oe-*HDAC1* group (CD180^–^ B lymphocytes transfected sh-*IRF1* and oe-*HDAC1*); oe-NC group (CD180^+^ B lymphocytes transfected with oe-NC), oe-*IRF1* group (CD180^+^ B lymphocytes transfected with oe-*IRF1*), oe-NC + sh-*HDAC1* group (CD180^+^ B lymphocytes transfected with oe-NC and sh-*HDAC1*), and oe-*IRF1* + sh-*HDAC1* group (CD180^+^ B lymphocytes transfected with oe-*IRF1* and sh-*HDAC1*).

In addition, the cells were further grouped into CD180^–^ B and CD180^+^ B groups. CD180 was overexpressed and divided into the oe-NC group (CD180^−^ B lymphocytes transfected with oe-NC) and the oe-*CD180* group (CD180^−^ B lymphocytes transfected with oe-*CD180*). To screen for the optimal concentration of lipopolysaccharide (LPS) treatment, we treated CD180^−^ B cells with different concentrations of LPS (0, 25, 50, 100, or 200 ng/mL; HY-D1056, MedChemExpress, Monmouth Junction, NJ, USA) and measured cell viability at 2, 4, and 8 hours post-treatment. CD180^−^ B lymphocytes were further treated with 100 ng/mL LPS (a TLR4 agonist) for 4 hours (Chistyakov et al., 2019), and divided into the oe-NC, oe-*CD180*, and oe-*CD180* + LPS groups. The knockdown of CD180 was divided into the sh-NC group (CD180^+^ B lymphocytes transfected with sh-NC) and the sh-CD180 group (CD180^+^ B lymphocytes transfected with sh-*CD180*). To screen for the optimal concentration of the TLR4 inhibitor TAK-242, we treated CD180^+^ B cells with different concentrations of TAK-242 (0, 0.25, 0.5, 1, or 2 μM; 243984-11-4, Sigma-Aldrich) for 1 hour and measured cell viability. CD180^+^ B lymphocytes were further treated with 1 μM of TAK-242 for 1 hour (Xie et al., 2019; Zhu et al., 2021) and divided into the sh-NC, sh-*CD180*, and sh-*CD180* + TAK-242 groups.

CD4^+^ T cells were stimulated with PMA (2 µg/mL) and ionomycin (5 µg/mL) for 4 hours. Next, CD180^−^ B cells transfected with oe-NC/oe*-CD180* were treated with or without the TLR4 agonist LPS (100 ng/mL) for 4 hours, or CD180^+^ B cells transfected with sh-NC/sh-*CD180* were treated with or without the TLR4 inhibitor TAK-242 (1 μM) for 1 hour, and B and T cells were then co-cultured. The experimental setups were as follows: Control group (CD180^−^ B lymphocytes cultured normally), oe-NC group (CD180^−^ B lymphocytes transfected with oe-NC), oe-*CD180* group (CD180^−^ B lymphocytes transfected with oe-*CD180*), and oe-*CD180* + LPS group (CD180^−^ B lymphocytes transfected with oe-*CD180* and treated with the TLR4 agonist LPS); Control group (CD180^+^ B lymphocytes cultured normally), sh-NC group (CD180^+^ B lymphocytes transfected with sh-NC), sh-*CD180* group (CD180^+^ B lymphocytes transfected with sh-*CD180*), and sh-*CD180* + TAK-242 group (CD180^+^ B lymphocytes transfected with oe-*CD180* and treated with the TLR4 inhibitor TAK-242). The flowchart of the experiment is shown in **Additional Figure 1**.

**Figure 1:**
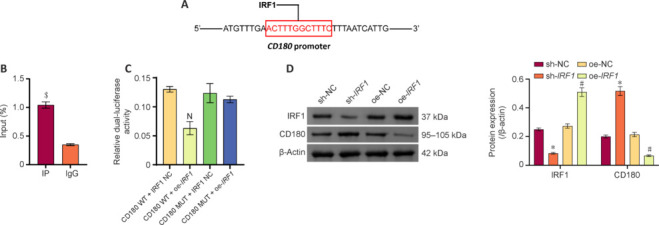
IRF1 inhibits CD180 transcription in MG. (A) A possible negative regulatory relationship between IRF1 and CD180 was predicted using the JASPAR2020 database. (B) ChIP determination of the binding of IRF1 and the *CD180* promoter. $*P* < 0.05, *vs*. IgG. (C) Dual-luciferase reporter gene assay revealed that IRF1 had transcriptional activity against the *CD180* promoter. ^N^*P* < 0.05, *vs.* CD180 WT + IRF1 NC. (D) Western blot detection of IRF1 and CD180 expression. *P < 0.05, *vs.* sh-NC; #*P* < 0.05, *vs.* oe-NC. Data are presented as the mean ± SD (*n* = 3), and were analyzed using the unpaired Student’s *t*-test (B) or one-way analysis of variance followed by Tukey’s *post hoc* test (C, D). ChIP: Chromatin immunoprecipitation; IRF1: interferon regulatory factor 1; MG: myasthenia gravis; WT: wild type.

The sh-*IRF1* (HG-SH002198), sh-*CD180* (HG-SH005582), and sh-*HDAC1* (HG-SH004964) RNA was obtained from HonorGene (Changsha, China). The sequences were as follows: sh-*IRF1* #1: 5′-GCT GGA CAG CCT GCT GAC CCC AGT CCG G-3′, sh-*IRF1* #2: 5′-GTT TGA GAC CAG CCT GGC CAA CAT GGT GA-3′, sh-*IRF1* #3: 5′-GCT CTG TCT GTA CCG TTC AAT ACA GTA GGC-3′; sh-*CD180* #1: 5′-GAC CCA AAA TGC TCT TCT CAT TTG GGC ATC CGG T-3′, sh-*CD180* #2: 5′-GCT CCT AGT TCT GCC ACC ATG CCT ATG CTG CTG AC-3′, sh-*CD180* #3: 5′-GCC TCC ATC ACA GAG GAC TCT CTG CAT GAG CTG C-3′; sh-*HDAC1* #1: 5′-GAA ATC TAT CGC CCT CAC AAA GCC AAT GCT G-3′, sh-*HDAC1* #2: 5′-GCA CGC ACC TGG GGT CCA AAT GCA GGC GAT T-3′, sh-*HDAC1* #3: 5′-GTG CCT GCT TAG TAG CTT TGG AAA GGT GCC CT-3′; sh-NC: 5′-GTA ATT AGC GTT TAA GGC CAA ATA CCG-3′. The oe-NC, oe-*IRF1* (HG-HO002198), oe-*CD180* (HG-HO005582), and oe-*HDAC1* (HG-HO004964) plasmids were also obtained from HonorGene.

### Quantitative reverse transcription-polymerase chain reaction

RNA was isolated from the T and B cells, followed by quantitative reverse transcription-polymerase chain reaction (PCR) to assess the levels of *IRF1*, *CD180*, *HDAC1*, *T-bet*, *GATA* binding protein 3 (*GATA3*), retinoic acid receptor-related orphan receptor gamma (*RORγ*), and forkhead box protein P3 (*FOXP3*). RNA extraction was performed using TRIzol reagent. A reverse transcription kit (CW2569, CWBIO, Taizhou, China) was used to synthesize complementary DNA from the isolated mRNA. The PCR reaction was set up using UltraSYBR Mixture (CW2601, CWBIO). Subsequently, a PCR detection system (QuantStudio1, Thermo Fisher Scientific, Waltham, MA, USA) was used to conduct the PCR and track the fluorescence signal. The 2^–ΔΔCt^ method was used to calculate gene expression (Rao et al., 2013), which was normalized to β*-actin* as a housekeeping gene. The primer sequences are outlined in **Table 1**.

**Table 1:** The primers in polymerase chain reaction

Gene	Sequences (5'–3')
*H-β-actin*	F: ACC CTG AAG TAC CCC ATC GAG
	R: AGC ACA GCC TGG ATA GCA AC
*H-IRF1*	F: CCA GAG CAG GAA CAA GGG
	R: GTG GTC ATC AGG CAG AGT G
*H-CD180*	F: CCA ATT TCC CCT CCC TTA CAC AC
	R: TTT CTC CAA GCA GCC AAC ACC
*H-T-bet*	F: ATG ATT GTG CTC CAG TCC CTC
	R: CCT CTG GCT CTC CGT CGT TC
*H-GATA3*	F: GTT GTG CTC GGA GGG TTT CT
	R: GCA CGC TGG TAG CTC ATA CA
*H-RORγ*	F: CAC CAG CCG AAA CCG ATG CC
	R: TCC CTC TGC TTC TTG GAC ATG CG
*H-Foxp3*	F: CGC CAC AAC CTG AGT CTG C
	R: CTC CAG CTC ATC CAC GGT CCA

Foxp3: Forkhead box protein P3; GATA3: GATA binding protein 3; IRF1: interferon regulatory factor 1; RORγ: retinoic acid receptor-related orphan receptor gamma.

### Western blot assay

Protein levels of IRF1, CD180, HDAC1, TLR4, p38 mitogen-activated protein kinase (p38MAPK), phosphorylated (p-)p38MAPK, extracellular signal-regulated kinase 1/2 (ERK1/2), p-ERK1/2, c-Jun N-terminal kinase (JNK), p-JNK, NF-κB p65, and p-NF-κB p65 in cellular extracts were analyzed using western blot analysis. Cellular or tissue total protein was extracted using radioimmunoprecipitation assay (P0013B, Beyotime, Shanghai, China), and protein concentrations were determined using a bicinchoninic acid protein assay kit (BL521A, Biosharp, Hefei, Anhui, China). Proteins were denatured at 100°C for 5 minutes and resolved using sodium dodecyl sulfate polyacrylamide gel electrophoresis. They were then transferred onto nitrocellulose membranes and blocked with 5% skim milk for 2 hours. Next, membranes were incubated with IRF1 (rabbit, 1:1000, Abcam, Cambridge, UK, Cat# ab230652, RRID: AB_2738487), CD180 (rabbit, 1:1000, Proteintech, Rosemont, IL, USA, Cat# 28304-1-AP, RRID: AB_2619861), HDAC1 (rabbit, 1:10,000, Proteintech, Cat# 10197-1-AP, RRID: AB_2619858), TLR4 (rabbit, 2 μg/mL, Abcam, Cat# ab13867, RRID: AB_776874), p38MAPK (rabbit, 1:2000, Abcam, Cat# ab170099, RRID: AB_2620058), p-p38MAPK (rabbit, 1:1000, Abcam, Cat# ab47363, RRID: AB_2241564), ERK1/2 (rabbit, 1:3000, Proteintech, Cat# 11257-1-AP, RRID: AB_2619863), p-ERK1/2 (rabbit, 1:5000, Proteintech, Cat# 28733-1-AP, RRID: AB_2619864), JNK (mouse, 1:20 000, Proteintech, Cat# 66210-1-Ig, RRID: AB_2619865), p-JNK (rabbit, 1:2000, Proteintech, Cat# 80024-1-RR, RRID: AB_2619866), NF-κB p65 (mouse, 1:1000, Proteintech, Cat# 66535-1-Ig, RRID: AB_2619867), p-NF-κB p65 (rabbit, 1:1000, Abcam, Cat# ab76302, RRID: AB_2241565), and β-actin (mouse, 1:5000, Proteintech, Cat# 66009-1-Ig, RRID: AB_2619868) overnight at 4°C. Horseradish peroxidase-conjugated secondary antibodies (goat anti-mouse IgG, 1:5000, Proteintech, Cat# SA00001-1, RRID: AB_2619869 or goat anti-rabbit IgG, 1:6000, Proteintech, Cat# SA00001-2, RRID: AB_2619870) were applied for 90 minutes at room temperature. Protein bands were then visualized using Super ECL Plus detection reagent (AWB0005, Abiowell, Changsha, China), with β-actin serving as an internal control.

### Flow cytometry

Flow cytometry analysis was conducted to quantify CD180^+^ and CD180^−^ B lymphocytes in peripheral blood. Cells were resuspended in 500 μL of RPMI-1640 medium supplemented with 10% fetal bovine serum. Cells were then centrifuged at 350 × *g* for 5 minutes, and the supernatant was removed. Next, anti-CD180 antibody (212911, BioLegend, San Diego, CA, USA) was added to the cells, mixed thoroughly, and incubated in the dark for 30 minutes. Following one wash with 1 mL of 0.5% bovine serum albumin-phosphate-buffered saline (PBS) and centrifugation at 400 × *g* for 5 minutes, the supernatant was aspirated. The cells were then resuspended in 150 μL of 0.5% bovine serum albumin-PBS for flow cytometric analysis (A00-1-1102, Beckman, Brea, CA, USA).

Flow cytometry was also used to detect the levels of CD19^+^CD27^+^ cells in B cells. Cells were collected and rinsed twice with PBS, and anti-CD19 (11-0199-42, eBioscience, San Diego, CA, USA) and anti-CD27 (12-0279-42, eBioscience) antibodies were introduced and incubated for 30 minutes in the dark. Cell precipitation was then collected by centrifugation, washed three times with staining buffer, resuspended in 200 μL of staining buffer, and subjected to detection.

The levels of CD4^+^CD40L^+^ cells in T cells were also determined using flow cytometry. Cells were collected, rinsed twice with PBS, and resuspended in 100 μL of basal medium. Subsequently, anti-CD4 (11-0041-82, eBioscience) and anti-CD40L (12-1548-42, eBioscience) antibodies were introduced and incubated for 30 minutes in the dark (with the establishment of negative control and single stain controls). Cells were washed with PBS twice, 200 μL of staining buffer was added, and the cells were analyzed using a flow cytometer.

### Cell apoptosis assessment

Processed cells were harvested using ethylenediaminetetraacetic acid-free pancreatic digestion enzymes. Subsequently, they were rinsed twice with PBS, centrifuged at 356 × *g* for 5 minutes each time, and the cell pellet was collected. Next, 500 μL of binding buffer was added to the cells, followed by 5 μL Annexin V-APC (8144, BioLegend) and 5 μL propidium iodide (00-6990-50, eBioscience); the mixture was then thoroughly combined. Subsequently, the cells were incubated for 10 minutes in the dark; they were then analyzed within 1 hour using a flow cytometer.

### Cell cycle assessment

First, 1 mL of chilled PBS was used to resuspend the cells, followed by centrifugation at 142.4 × *g* for 5 minutes to pellet the cells, after which the supernatant was aspirated. The cells were then gently suspended in 400 μL of PBS to create a single-cell suspension. Next, 1.2 mL of pre-chilled 100% ethanol was gradually added to achieve a 75% final concentration, and the cells were fixed in this solution at 4°C overnight. The fixed cells were collected and centrifuged at 142.4 × *g* for 5 minutes, and the supernatant was discarded. Next, cells were washed with 1 mL of cold PBS and centrifuged again at 142.4 × *g* for 5 minutes. The cell pellet was then resuspended in 150 μL of propidium iodide (PI) staining solution and incubated at 4°C for 30 minutes. Flow cytometry analysis was conducted, with PI excitation by a 488 nm argon ion laser and detection through a 630 nm filter. A total of 10,000 events were collected based on the forward scatter/side scatter plot. A gating strategy was used to exclude debris and clumped cells, and the cell cycle distribution was analyzed from the PI fluorescence histogram.

### Cell counting kit-8 assay

The cells were digested and counted before being seeded onto a 24-well plate at a density of 1 × 10^4^ cells per well, with 300 μL of complete culture medium per well. After the cells adhered to the walls of the wells, they were treated with either different concentrations of LPS (0, 25, 50, 100, or 200 ng/mL; HY-D1056, MedChemExpress) for 2, 4, and 8 hours post-treatment or different concentrations of the TLR4 inhibitor TAK-242 (0, 0.25, 0.5, 1, or 2 μM; 243984-11-4, Sigma-Aldrich) for 1 hour. Next, 30 μL of cell counting kit-8 (CCK-8) solution (CK001, Dojindo, Kumamoto, Japan) was added to each well. The culture medium containing the drug was removed, and 300 μL of fresh culture medium was added to each well. After incubation at 37°C with 5% CO_2_ for another 4 hours, the optical density at 450 nm was analyzed using a microplate reader (BioTek, Winooski, VT, USA).

### Enzyme-linked immunosorbent assay

Enzyme-linked immunosorbent assay was conducted to measure the concentrations of IFN-γ, interleukin (IL)-4, IL-17, and TGF-β1. For this purpose, specific quantitative enzyme-linked immunosorbent assay kits for IFN-γ (KE00146, Proteintech), IL-4 (KE00232, Proteintech), IL-17 (KE00203, Proteintech), and TGF-β1 (KE00002, Proteintech) were used following the manufacturer’s guidelines.

### Dual-luciferase reporter gene assay

To confirm the interaction between *IRF1* and *CD180*, wild-type or mutant *CD180* DNA fragments were cloned into pGL3 vectors. The recombinant plasmids were introduced into cells using Lipofectamine 2000, following the manufacturer’s protocol. *IRF1* NC (5′-GGT GAG AGA GAA AGT-3′, HonorGene) and oe-*IRF1* (5′-CCA CTC TCT CTT TCA-3′, HonorGene) were co-transfected. In parallel, *CD180* promoter-driven luciferase reporters were transfected into B cells in the presence of IRF1 and individual HDACs (HDAC1, HDAC2, HDAC3, HDAC4, HDAC5, HDAC7, or HDAC11) to identify the HDAC isoform that most potently repressed *CD180* promoter activity. To ascertain the association between HDAC1 and *IRF1*, wild-type or mutant *IRF1* fragments were inserted into pGL3 vectors (E1910, Promega, Madison, WI, USA), and oe-*IRF1* along with oe-*HDAC1* were co-transfected into cells. Luciferase activity was then assessed using a Nano-Glo dual-luciferase assay system (N1620, Promega).

### Chromatin immunoprecipitation

A possible negative regulatory relationship between IRF1 and *CD180* has been predicted using the JASPAR2020 database (https://jaspar.genereg.net/; Rauluseviciute et al., 2024). In accordance with the chromatin immunoprecipitation (ChIP) kit (P2078, Beyotime) guidelines, the binding of IRF1 (sc-514544) or HDAC1 to the CD180 promoter region was examined. Cells were fixed with 1% formaldehyde for 10 minutes, followed by sonication to generate DNA fragments ranging from 200–800 bp. The DNA fragments were then immunoprecipitated using antibodies specific to IRF1. Subsequently, ChIP DNA was purified and eluted with 100 μL of H_2_O, and 2.5 μL of this DNA was used for quantitative reverse transcription PCR analysis (Wang et al., 2020). This process allowed for the detection of IRF1 enrichment at the *CD180* gene promoter.

### Co-immunoprecipitation

Co-immunoprecipitation was conducted to ascertain the association between HDAC1 and IRF1 in B lymphocytes. The HDAC1 antibody served as the bait, and the levels of HDAC1 and IRF1 post-co-immunoprecipitation were analyzed using western blot analysis. Initially, the cells were harvested and processed for protein extraction. Next, anti-HDAC1 antibody and normal rabbit IgG (B900610, Proteintech) were allowed to react overnight at 4°C. Subsequently, agarose bead coupling was conducted. The precipitated agarose beads were then mixed with the immunoprecipitation lysate for western blot analysis. The primary antibodies used were anti-HDAC1 (1:10,000) and anti-IRF1 (1:1000).

### Statistical analysis

Statistical analysis was conducted using GraphPad Prism (version 10.0.0 for Windows, GraphPad Software, Boston, MA, USA, www.graphpad.com). Data are presented as the mean ± standard deviation. The Kolmogorov–Smirnov test and exploratory descriptive statistical tests were used to assess whether the data followed a normal distribution and exhibited homogeneity of variance. The measurement data obeyed a normal distribution and homogeneity of variance. The data were then analyzed using parametric tests. Specifically, the unpaired Student’s *t*-test was applied for comparisons between two non-paired groups. For comparisons across multiple groups, one-way analysis of variance was conducted followed by Tukey’s *post hoc* test. Data comparisons between groups at different time points were analyzed using two-way analysis of variance with the Bonferroni *post hoc* test. *P* < 0.05 was considered to denote significance.

## Results

### Interferon regulatory factor 1 inhibits CD180 transcription in B cells in myasthenia gravis

Our prior research indicates that compared with healthy subjects, patients with MG exhibit reduced CD180 expression and have a greater presence of CD180^−^ B cells (Jin et al., 2021). Transcriptome sequencing of B cells from patients with MG compared with healthy individuals revealed that the transcription factor IRF1 is overexpressed and plays a role in leukocyte differentiation and activation. In the present investigation, we aimed to further elucidate the possible molecular mechanisms through which IRF1 influences CD180^−^ B cells in MG. JASPAR2020 database prediction demonstrated that IRF1 and *CD180* may have a negative regulatory relationship (**Figure 1A**). ChIP experiments further determined the binding of IRF1 and *CD180* promoters (**Figure 1B**). In addition, the promoter activity of IRF1 on *CD180* was validated using a dual-luciferase reporter assay (**Figure 1C**). We then targeted *IRF1* for knockdown. Compared with the sh-NC group, IRF1 expression was lower in the sh-*IRF1* #1, sh-*IRF1* #2, and sh-*IRF1* #3 groups. Notably, the sh-*IRF1* #3 group exhibited most substantial decrease in IRF1 expression, leading to the selection of sh-*IRF1* #3 for subsequent research (**Additional Figure 1A**). Furthermore, *IRF1* interference inhibited IRF1 expression and promoted CD180 expression. Conversely, *IRF1* overexpression promoted IRF1 expression and inhibited CD180 expression (**Figure 1D**). Together, these results suggest that IRF1 inhibits CD180 transcription.

### Interferon regulatory factor 1 promotes B cell activation in myasthenia gravis

**Figure 2:**
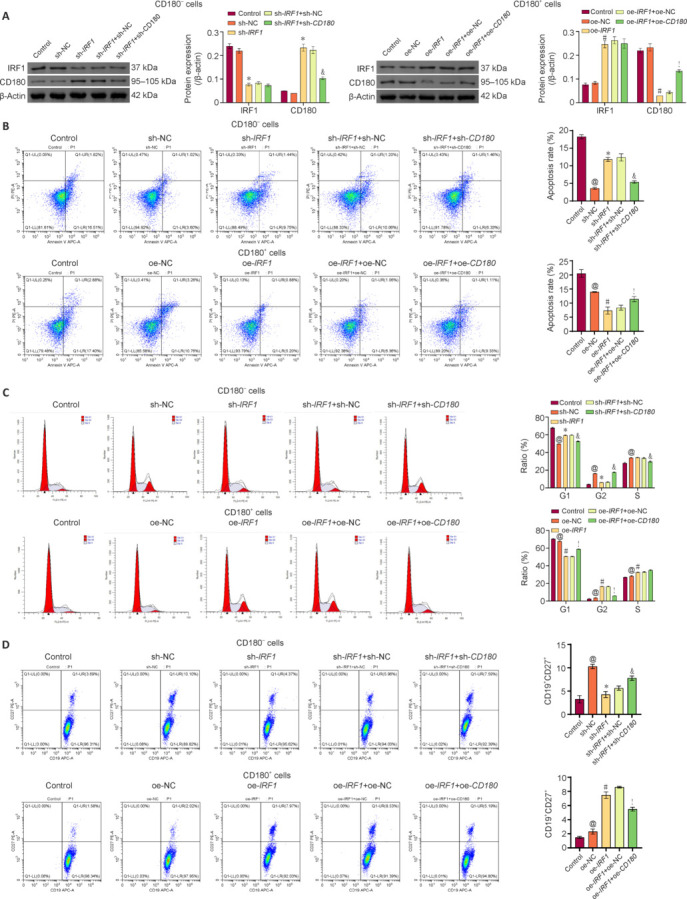
IRF1 promotes B cell activation in MG. (A) Assessment of IRF1 and CD180 protein levels in B cells using western blot analysis. *P < 0.05, *vs.* sh-NC; &*P* < 0.05, *vs.* sh-*IRF1* + sh-NC; #*P* < 0.05, *vs.* oe-NC; !*P* < 0.05, *vs*. oe-*IRF1* + oe-NC. (B) Measurement of B cell apoptosis rates using flow cytometry. (C) Analysis of the B cell cycle phase distribution using flow cytometry. (D) Analysis of the levels of CD19^+^CD27^+^ in B cells using flow cytometry. @*P* < 0.05, *vs*. Control; *P < 0.05, *vs.* sh-NC; &*P* < 0.05, *vs.* sh-*IRF1* + sh-NC; #*P* < 0.05, *vs.* oe-NC; !*P* < 0.05, *vs*. oe-*IRF1* + oe-NC. Data are presented as the mean ± SD (*n* = 3) and were analyzed using one-way analysis of variance followed by Tukey’s *post hoc* test. IRF1: Interferon regulatory factor 1; MG: myasthenia gravis.

To investigate the effects of IRF1 on B cell activation, we used magnetic bead separation to isolate peripheral blood T cells, conducted co-cultures with peripheral blood B cells, and knocked down CD180. Relative to the sh-NC group, CD180 expression was diminished in the sh-*CD180* #1, sh-*CD180* #2, and sh-*CD180* #3 groups; the sh-*CD180* #3 group exhibited the most notable reduction, prompting the selection of sh-*CD180* #3 for further investigations (**Additional Figure 1B**). Compared with the sh-NC group, the sh-*IRF1* group exhibited reduced IRF1 expression and elevated CD180 expression. Upon subsequent *CD180* knockdown, IRF1 levels remained largely unchanged, whereas CD180 expression decreased. Compared with the oe-NC group, the oe-*IRF1* group showed increased IRF1 expression and decreased CD180 expression. Following *CD180* overexpression, IRF1 levels were relatively stable, whereas CD180 expression rose (**Figure 2A**). Furthermore, compared with the sh-NC group, B cell apoptosis was higher in the sh-*IRF1* group, with an increase in the G1 phase and a decrease in the G2 + S phase. Following additional *CD180* knockdown, B cell apoptosis was reduced, the G1 phase was shorter, and the G2 + S phase was longer. Compared with the oe-NC group, the oe-*IRF1* group exhibited reduced B cell apoptosis, a shorter G1 phase, and a longer G2 + S phase. After *CD180* overexpression, B cell apoptosis rose, the G1 phase extended, and the G2 + S phase shortened (**Figure 2B** and **C**, and **Additional Figure 2A**). Additionally, compared with the sh-NC group, the expression of CD19^+^CD27^+^ in B cells was reduced in the sh-*IRF1* group. After further knockdown of CD180, CD19^+^CD27^+^ cells increased. Compared with the oe-NC group, the level of CD19^+^CD27^+^ cells in B cells was increased in the oe-*IRF1* group. After CD180 overexpression, the level of CD19^+^CD27^+^ cells in B cells decreased (**Figure 2D** and **Additional Figure 2B**). Collectively, our findings indicate that IRF1 enhances B cell activation.

### Interferon regulatory factor 1 promotes T cell differentiation by inhibiting CD180 in myasthenia gravis

**Figure 3:**
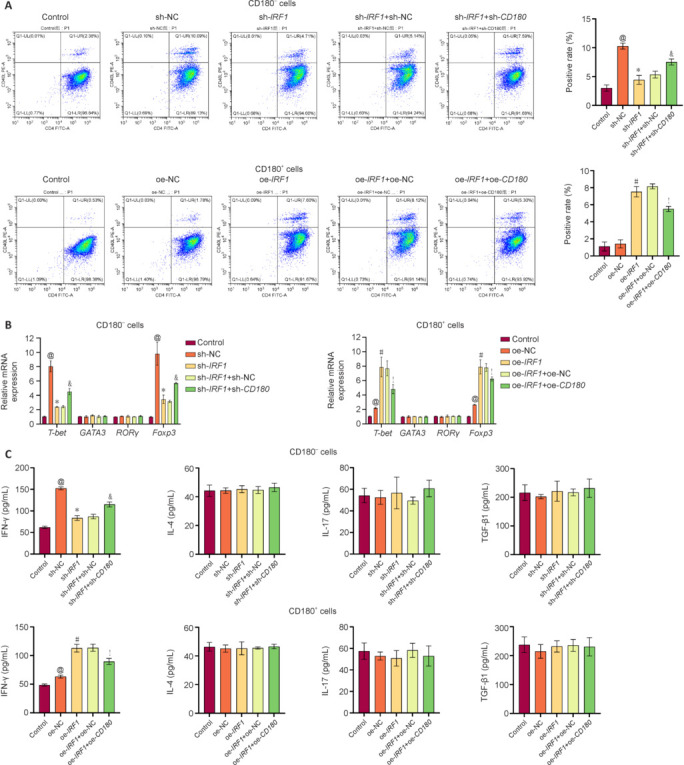
IRF1 promotes T cell differentiation by inhibiting CD180 in MG. (A) Analysis of CD4^+^CD40L^+^ expression in T cells using flow cytometry. (B) Quantitative reverse transcription-polymerase chain reaction was used to measure *T-bet*, *GATA3*, *RORγ*, and *Foxp3* levels in T cells. (C) Enzyme linked immunosorbent assay was used to determine IFN-γ, IL-4, IL-17, and TGF-β1 concentrations in supernatants from T cell cultures. @*P* < 0.05, *vs*. Control; *P < 0.05, *vs.* sh-NC; &*P* < 0.05, *vs.* sh-IRF1 + sh-NC; #*P* < 0.05, *vs.* oe-NC; !*P* < 0.05, *vs.* oe-IRF1 + oe-NC. Data are presented as the mean ± SD (*n* = 3) and were analyzed using one-way analysis of variance followed by Tukey’s *post hoc* test. Foxp3: Forkhead box protein P3; GATA3: GATA binding protein 3; IFN: type I interferon; IL: interleukin; IRF1: interferon regulatory factor 1; MG: myasthenia gravis; RORγ: retinoic acid receptor-related orphan receptor gamma; TGF-β1: transforming growth factor β1.

To explore the role of IRF1 in T cell differentiation, we examined the expression of relevant indicators in T cells. Compared with the sh-NC group, the sh-*IRF1* group exhibited reduced levels of CD4^+^CD40L^+^ cells, T-bet, and FOXP3 in T cells. However, upon additional CD180 knockdown, the levels of CD4^+^CD40L^+^ cells, T-bet, and FOXP3 increased. The oe-*IRF1* group exhibited higher expression of CD4^+^CD40L^+^ cells, T-bet, and FOXP3 in T cells than the oe-NC group. Conversely, CD180 overexpression reversed the expression of these markers. Nevertheless, there were no significant differences in GATA3 or RORγ levels across all groups (**Figure 3A** and **B** and **Additional Figure 2C**). Moreover, the sh-IRF1 group had lower T cell supernatant IFN-γ levels than the sh-NC group. This was reversed with additional *CD180* knockdown, as IFN-γ levels increased. The oe-IRF1 group also had higher IFN-γ levels than the oe-NC group; however, *CD180* overexpression resulted in decreased IFN-γ levels. By contrast, IL-4, IL-17, and transforming growth factor β1 (TGF-β1) levels remained largely unchanged among all groups (**Figure 3C**). Our results suggest that IRF1 promotes T cell differentiation by inhibiting CD180.

### Interferon regulatory factor 1 recruits histone deacetylase 1 to inhibit CD180 transcription in B cells of myasthenia gravis

**Figure 4:**
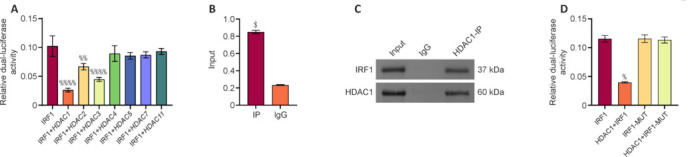
IRF1 recruits HDAC1 to inhibit CD180 transcription in MG. (A) *CD180* promoter luciferase constructs were transfected into B cells with IRF1 and different HDACs (overexpressing *HDAC1*, *HDAC2*, *HDAC3*, *HDAC4*, *HDAC5*, *HDAC7*, or *HDAC11*). HDAC1 most effectively inhibited CD180 promoter activity. %%*P* < 0.01, %%%%*P* < 0.0001, *vs*. IRF1. (B) ChIP assay with anti-HDAC1 or IgG confirmed that HDAC1 was recruited to the *CD180* promoter region of B cells. $*P* < 0.05, *vs.* IgG. (C) Co-immunoprecipitation confirmed that HDAC1 interacted directly with IRF1 in B cells. (D) Dual-luciferase experiments confirmed that HDAC1 inhibited the activity of the CD180 promoter containing the IRF1 site. %*P* < 0.05, *vs.* IRF1. Data are presented as the mean ± SD (*n* = 3), and were analyzed using the unpaired Student’s *t*-test (B) or one-way analysis of variance followed by Tukey’s *post hoc* test (A and D). HDAC1: Histone deacetylase 1; IP: immunoprecipitation; IRF1: interferon regulatory factor 1; MG: myasthenia gravis.

To elucidate the mechanisms by which IRF1 and HDAC1 affect *CD180* transcription, we transfected *CD180* promoter luciferase constructs into B cells with IRF1 and different HDACs (to overexpress *HDAC1*, *HDAC2*, *HDAC3*, *HDAC4*, *HDAC5*, *HDAC7*, or *HDAC11*). HDAC1 most effectively inhibited *CD180* promoter activity (**Figure 4A**). ChIP further confirmed that HDAC1 was recruited to the CD180 promoter region of B cells (**Figure 4B**). Co-immunoprecipitation demonstrated that HDAC1 interacted directly with IRF1 in B cells (**Figure 4C**). Finally, dual-luciferase experiments confirmed that HDAC1 was able to inhibit the activity of the *CD180* promoter containing the IRF1 site, and that this ability was lost when the IRF1 site was mutated (**Figure 4D**). Together, our results suggest that IRF1 recruits HDAC1 to inhibit *CD180* transcription.

### Histone deacetylase 1 promotes B cell activation in myasthenia gravis

**Figure 5:**
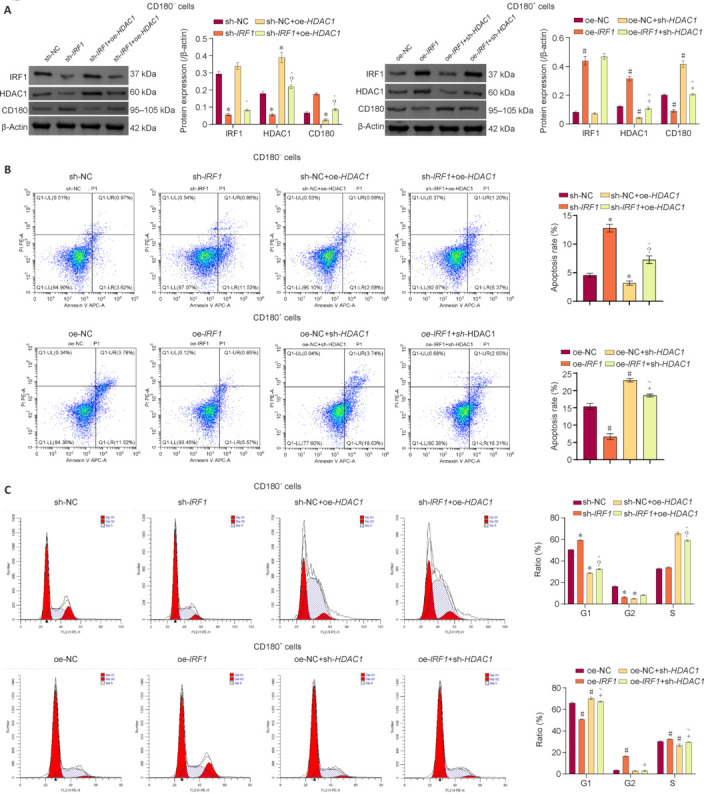
HDAC1 promotes B cell activation in MG. (A) Western blot analysis of IRF1, CD180, and HDAC1 expression. (B) B cell apoptosis was analyzed using flow cytometry. (C) B cell cycle was measured using flow cytometry. Data are presented as the mean ± SD (*n* = 3) and were analyzed using one-way analysis of variance followed by Tukey’s *post hoc* test. *P < 0.05, *vs.* sh-NC; ^*P* < 0.05, *vs.* sh-*IRF1*; ?*P* < 0.05, *vs.* sh-NC + oe-*HDAC1*; #*P* < 0.05, *vs*. oe-NC; ~*P* < 0.05, *vs.* oe-*IRF1*; +*P* < 0.05, *vs.* oe-NC + sh-*HDAC1*. HDAC1: Histone deacetylase 1; IRF1: interferon regulatory factor 1; IL: interleukin; MG: myasthenia gravis; IFN: type I interferon.

To investigate the effects of HDAC1 on B cell activation, we conducted experiments to knock down HDAC1. Compared with the sh-NC group, the sh-*HDAC1* #1, sh-*HDAC1* #2, and sh-*HDAC1* #3 groups exhibited reduced HDAC1 expression. Notably, the sh-*HDAC1* #3 group had the most pronounced reduction in HDAC1 expression, leading to its selection for subsequent analyses (**Additional Figure 3**). Relative to the sh-NC group, the sh-IRF1 group displayed decreased IRF1 and HDAC1 expression and increased CD180 expression. When *IRF1* was knocked down in conjunction with *HDAC1* overexpression, both IRF1 and HDAC1 levels decreased, whereas CD180 expression was elevated. In contrast to the oe-NC group, the oe-*IRF1* group had increased IRF1 and HDAC1 expression as well as decreased CD180 expression. When *IRF1* was overexpressed following *HDAC1* knockdown, both IRF1 and HDAC1 levels increased and CD180 expression was reduced (**Figure 5A**). Furthermore, compared with the sh-NC group, the sh-*IRF1* group displayed higher B cell apoptosis, a longer G1 phase, and a shorter G2 + S phase. When *IRF1* was knocked down following *HDAC1* overexpression, B cell apoptosis decreased, the G1 phase was shorter, and the G2 + S phase was longer. Compared with the oe-NC group, the oe-*IRF1* group exhibited reduced B cell apoptosis, a shorter G1 phase, and a longer G2 + S phase. Conversely, when IRF1 was overexpressed after *HDAC1* knockdown, B cell apoptosis increased, the G1 phase lengthened, and the G2 + S phase shortened (**Figure 5B** and **C** and **Additional Figure 4A**). Collectively, our results indicate that HDAC1 promotes B cell activation.

### Interferon regulatory factor 1 promotes T cell differentiation through histone deacetylase 1 in myasthenia gravis

**Figure 6:**
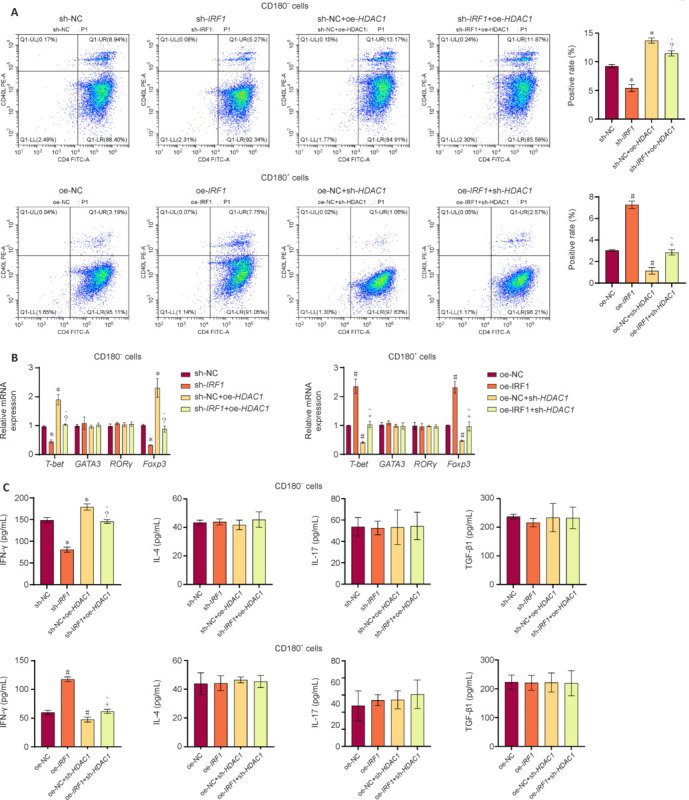
IRF1 promotes T cell differentiation through HDAC1 in MG. (A) Flow cytometry of CD4^+^CD40L^+^ in T cells. (B) *T-bet*, *GATA3*, *RORγ*, and *Foxp3* mRNA expression in T cells were quantified using quantitative reverse transcription-polymerase chain reaction. (C) IFN-γ, IL-4, IL-17, and TGF-β1 levels in T cell supernatants were analyzed using enzyme linked immunosorbent assay. Data are presented as the mean ± SD (*n* = 3) and were analyzed using one-way analysis of variance followed by Tukey’s *post hoc* test. *P < 0.05, *vs.* sh-NC; ^*P* < 0.05, *vs.* sh-*IRF1*; ?*P* < 0.05, *vs.* sh-NC + oe-*HDAC1*; #*P* < 0.05, *vs.* oe-NC; **~***P* < 0.05, *vs.* oe-*IRF1*; +*P* < 0.05 *vs.* oe-NC + sh-*HDAC1*. Foxp3: Forkhead box protein P3; GATA3: GATA binding protein 3; HDAC1: histone deacetylase 1; IFN: type I interferon; IL: interleukin; IRF1: interferon regulatory factor 1; MG: myasthenia gravis; RORγ: retinoic acid receptor-related orphan receptor gamma; TGF-β1: transforming growth factor β1.

To explore the role of IRF1 in promoting T cell differentiation, we examined the expression of relevant indicators in T cells. Compared with the sh-NC group, the sh-*IRF1* group displayed reduced levels of CD4^+^CD40L^+^, T-bet, and FOXP3 in T cells. However, when *IRF1* was knocked down following *HDAC1* overexpression, CD4^+^CD40L^+^, T-bet, and FOXP3 levels increased in T cells. The oe-*IRF1* group exhibited higher levels of CD4^+^CD40L^+^ cells, T-bet, and FOXP3 in T cells than the oe-NC group. Conversely, when *IRF1* was overexpressed after *HDAC1* knockdown, the expression of these markers decreased. There were no significant differences in GATA3 or RORγ levels across all groups (**Figure 6A** and **B** and **Additional Figure 4B**). Furthermore, the sh-*IRF1* group had lower IFN-γ levels in T cell supernatants than the sh-NC group. Knocking down *IRF1* following *HDAC1* overexpression resulted in increased IFN-γ levels. The oe-*IRF1* group also had higher IFN-γ levels than the oe-NC group; however, overexpressing *IRF1* after *HDAC1* knockdown led to decreased IFN-γ levels. By contrast, IL-4, IL-17, and TGF-β1 levels remained largely unchanged among all groups (**Figure 6C**). Our results suggest that IRF1 promotes T cell differentiation through HDAC1.

### CD180 inhibits B cell activation and T cell differentiation by inhibiting the Toll-like receptor 4/mitogen-activated protein kinases/nuclear factor-kappa B pathway in myasthenia gravis

**Figure 7:**
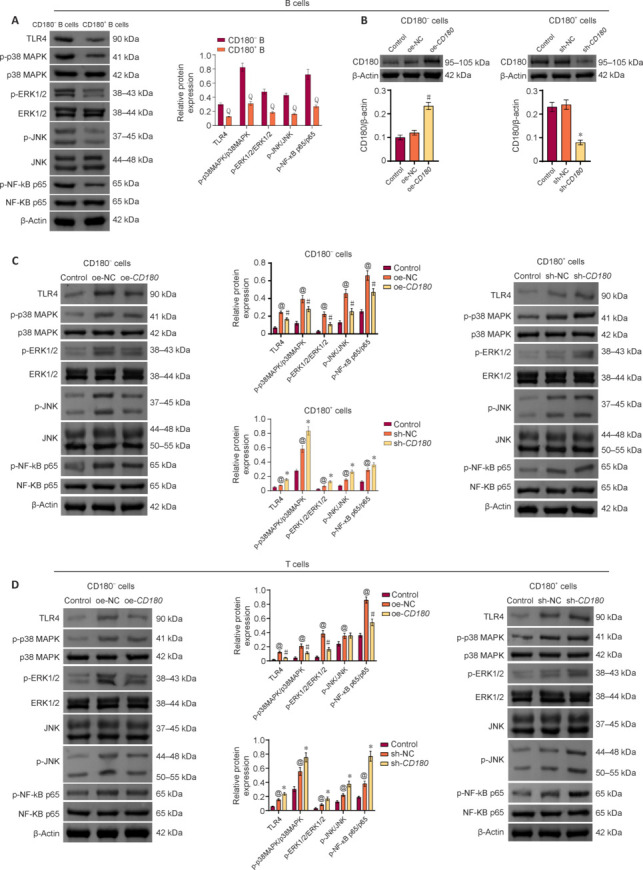
CD180 negatively regulates the TLR4/MAPKs/NF-κB pathway in B and T cells. (A) Western blot analysis of TLR4, p38MAPK, p-p38MAPK, ERK1/2, p-ERK1/2, JNK, p-JNK, NF-κB p65, and p-NF-κB p65 expression in CD180^−^ B cells and CD180^+^ B cells. ^Q^*P* < 0.05, *vs.* CD180^−^ B cells. (B) Western blot analysis of CD180 expression in B cells. (C) Western blot analysis of TLR4, p38MAPK, p-p38MAPK, ERK1/2, p-ERK1/2, JNK, p-JNK, NF-κB p65, and p-NF-κB p65 expression in B cells. (D) Western blot analysis of TLR4, p38MAPK, p-p38MAPK, ERK1/2, p-ERK1/2, JNK, p-JNK, NF-κB p65, and p-NF-κB p65 expression in T cells. @*P* < 0.05, *vs*. Control; #*P* < 0.05, *vs.* oe-NC; *P < 0.05, *vs.* sh-NC. ERK1/2: Extracellular signal-regulated kinase 1/2; JNK: c-Jun N-terminal kinase; MAPKs: mitogen-activated protein kinases; NF-κB p65: nuclear factor kappa B p65; NF-κB: nuclear factor-kappaB; p-ERK1/2: phosphorylated ERK1/2; p-JNK: phosphorylated JNK; p-NF-κB p65: phosphorylated NF-κB p65; p38MAPK: p38 mitogen-activated protein kinase; p-p38MAPK: phosphorylated p38MAPK; TLR4: Toll-like receptor 4.

To investigate the effects of CD180 on B cell activation and T cell differentiation via inhibition of the TLR4/MAPKs/NF-κB signaling pathway, we analyzed the expression of proteins related to the TLR4/MAPKs/NF-κB signaling pathway in CD180^−^ B and CD180^+^ B cells from patients with MG following plasma exchange therapy. CD180^+^ B cells had reduced TLR4 levels and phosphorylated-to-total ratios of p38MAPK, ERK1/2, JNK, and NF-κB p65 compared with CD180^−^ B cells (**Figure 7A**). Furthermore, CD180^−^ B cells in the oe-*CD180* group exhibited higher CD180 expression than those in the oe-NC group. By contrast, CD180^+^ B cells in the sh-*CD180* group showed decreased CD180 expression compared with the sh-NC group (**Figure 7B**). Additionally, CD180^−^ B cells in the oe*-CD180* group displayed reduced levels of phosphorylated-to-total p38MAPK, ERK1/2, JNK, and NF-κB p65 and decreased TLR4 levels compared with the oe-NC group. Conversely, CD180^+^ B cells in the sh-*CD180* group had increased levels of these proteins compared with the sh-NC group (**Figure 7C**). Consistent with this finding, CD180^−^ T cells in the oe-*CD180* group exhibited reduced levels of phosphorylated-to-total p38MAPK, ERK1/2, JNK, and NF-κB p65 and decreased TLR4 levels compared with the oe-NC group. Conversely, CD180^+^ T cells in the sh-*CD180* group had increased levels of these proteins compared with the sh-NC group (**Figure 7D**). To screen for the optimal concentration of LPS treatment, we treated CD180^−^ B cells with different concentrations of LPS (0, 25, 50, 100, or 200 ng/mL) and measured cell viability at 2, 4, and 8 hours. Cell viability was maintained at a relatively high level with an LPS concentration of 100 ng/mL (**Additional Figure 5A**). To screen for the optimal concentration of TLR4 inhibitor TAK-242 treatment, we treated CD180^+^ B cells with different concentrations of TAK-242 (0, 0.25, 0.5, 1, or 2 μM) for 1 hour and measured cell viability. Cell viability was maintained at a relatively high level with a TAK-242 concentration of 1 μM (**Additional Figure 5B**).

**Figure 8:**
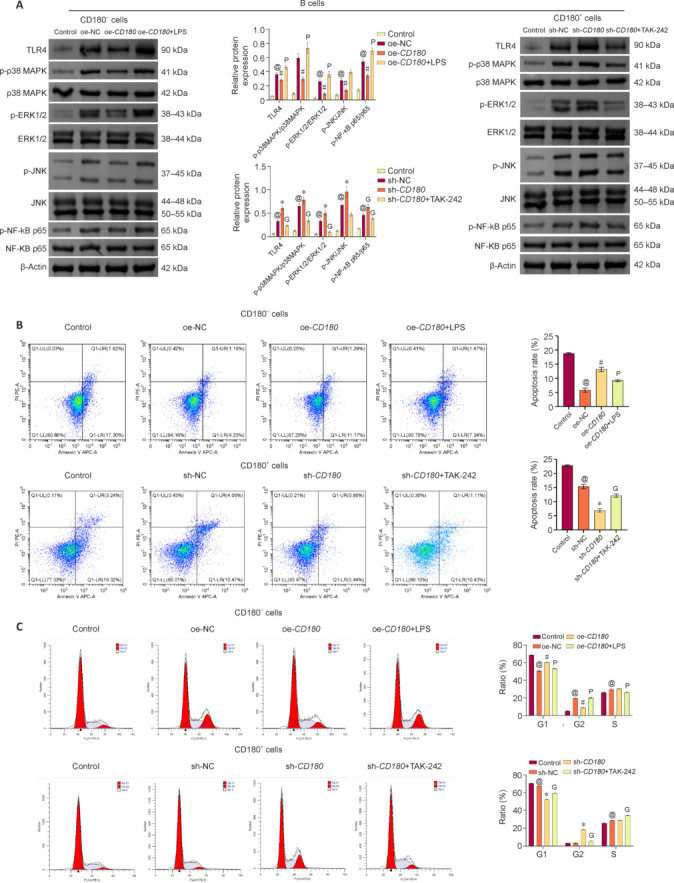
CD180 modulates B cell proliferation and apoptosis. (A) Western blot analysis of TLR4, p38MAPK, p-p38MAPK, ERK1/2, p-ERK1/2, JNK, p-JNK, NF-κB p65, and p-NF-κB p65 expression in B cells. (B) B cell apoptosis was measured using flow cytometry. (C) B cell cycle was determined using flow cytometry. @*P* < 0.05, *vs*. Control; #*P* < 0.05, *vs.* oe-NC; *P < 0.05, *vs*. sh-NC; ^P^*P* < 0.05, *vs*. oe-*CD180*; ^G^*P* < 0.05, *vs*. sh-*CD180*. Data are presented as the mean ± SD (*n* = 3), and were analyzed using one-way analysis of variance followed by Tukey’s *post hoc* test. ERK1/2: Extracellular signal-regulated kinase 1/2; JNK: c-Jun N-terminal kinase; MAPKs: mitogen-activated protein kinases; NF-κB: nuclear factor-kappaB; p-ERK1/2: phosphorylated ERK1/2; p-JNK: phosphorylated JNK; p-NF-κB p65: phosphorylated NF-κB p65; p38MAPK: p38 mitogen-activated protein kinase; p-p38MAPK: phosphorylated p38MAPK; TLR4: Toll-like receptor 4.

**Figure 9:**
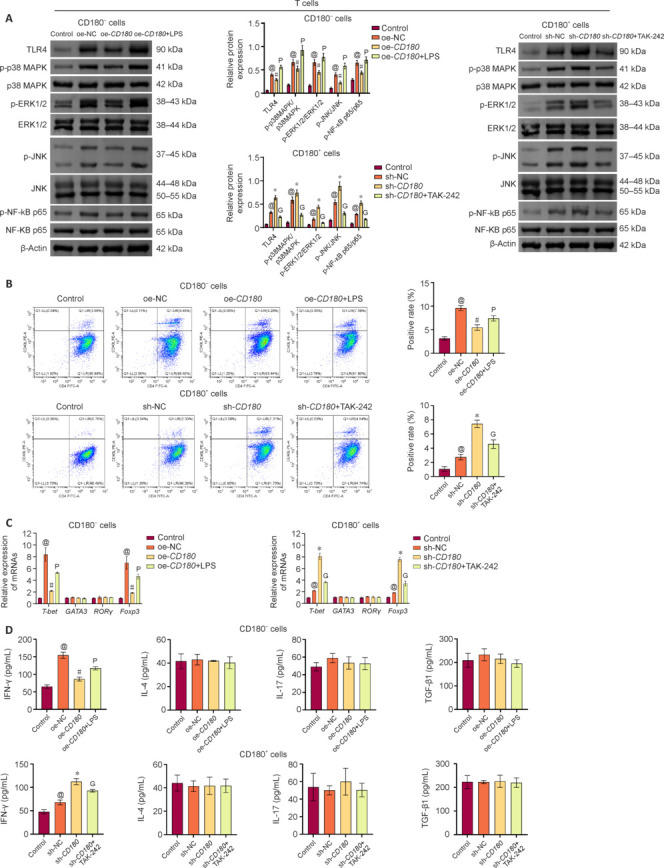
CD180 suppresses T cell activation and differentiation. (A) Western blot analysis of TLR4, p38MAPK, p-p38MAPK, ERK1/2, p-ERK1/2, JNK, p-JNK, NF-κB p65, and p-NF-κB p65 expression in T cells. (B) Flow cytometry of CD4^+^CD40L^+^ in T cells. (C) Levels of *T-bet*, *GATA3*, *RORγ*, and *Foxp3* in T cells were quantified using quantitative reverse transcription-polymerase chain reactionn. (D) IFN-γ, IL-4, IL-17, and TGF-β1 levels in T cell supernatants were determined using enzyme linked immunosorbent assay. @*P* < 0.05, *vs*. Control; #*P* < 0.05, *vs*. oe-NC; *P < 0.05, *vs*. sh-NC; ^P^*P* < 0.05, *vs.* oe-*CD180*; ^G^*P* < 0.05, *vs.* sh-*CD180*. Data are presented as the mean ± SD (*n* = 3), and were analyzed using one-way analysis of variance followed by Tukey’s *post hoc* test. ERK1/2: Extracellular signal-regulated kinase 1/2; Foxp3: forkhead box protein P3; GATA3: GATA binding protein 3; IL: interleukin; JNK: c-Jun N-terminal kinase; MAPKs: mitogen-activated protein kinases; NF-κB p65: nuclear factor kappa B p65; NF-κB: nuclear factor-kappaB; p-ERK1/2: phosphorylated ERK1/2; p-JNK: phosphorylated JNK; p-NF-κB p65: phosphorylated NF-κB p65; p38MAPK: p38 mitogen-activated protein kinase; p-p38MAPK: phosphorylated p38MAPK; RORγ: retinoic acid receptor-related orphan receptor gamma; TLR4: Toll-like receptor 4.

Furthermore, CD4^+^ T cells from patients with MG were activated with PMA and ionomycin for 4 hours, followed by co-culturing with oe-NC/oe-*CD180*-transfected CD180^−^ B cells with or without the TLR4 agonist LPS, and with sh-NC/sh-*CD180*-transfected CD180^+^ B cells with or without the TLR4 inhibitor TAK-242. When we treated CD180^−^ B and CD180^+^ B cells with LPS and TAK-242, respectively, we observed that CD180^−^ B cells in the oe-*CD180* group had decreased levels of TLR4 and the aforementioned proteins (phosphorylated-to-total p38MAPK, ERK1/2, JNK, and NF-κB p65) compared with the oe-NC group. However, the addition of LPS led to an increase in these protein levels. Similarly, CD180^+^ B cells in the sh-*CD180* group had increased levels of TLR4 and the other proteins, which were reduced upon treatment with TAK-242 (**Figure 8A**). Relative to the oe-NC group, CD180^−^ B cells in the oe-*CD180* group exhibited increased apoptosis, an extended G1 phase, and a reduced G2 + S phase. However, the addition of LPS led to a decrease in apoptosis, a shortened G1 phase, and an extended G2 + S phase. By contrast, CD180^+^ B cells in the sh-*CD180* group showed decreased apoptosis, a shortened G1 phase, and an extended G2 + S phase compared with the sh-NC group, and these trends were reversed by TAK-242 treatment (**Figure 8B** and **C** and **Additional Figure 6B**). Moreover, we observed that CD180^−^ T cells in the oe-*CD180* group had decreased levels of TLR4 and the aforementioned proteins (phosphorylated-to-total p38MAPK, ERK1/2, JNK, and NF-κB p65) compared with the oe-NC group. However, the addition of LPS led to an increase in these protein levels. Similarly, CD180^+^ T cells in the sh-*CD180* group had increased levels of TLR4 and the other proteins, and these levels were reduced upon treatment with TAK-242 (**Figure 9A**). CD4^+^CD40L^+^, T-bet, and FOXP3 levels in T cells were reduced in the oe-CD180 group compared with the oe-NC group, but were increased with the addition of LPS. Similarly, these levels were higher in the sh-*CD180* group compared with the sh-NC group, and decreased after TAK-242 treatment. There were no significant changes in GATA3 or RORγ levels (**Figure 9B** and **C** and **Additional Figures 6B** and **7**). The oe-*CD180* group had lower IFN-γ levels in T cell supernatants than the oe-NC group, and these levels increased with LPS addition. The sh-CD180 group also had higher IFN-γ levels than the sh-NC group, and these levels decreased after TAK-242 treatment. There were no significant changes in IL-4, IL-17, or TGF-β1 levels (**Figure 9D**). Collectively, our results indicate that *CD180* inhibits B cell activation and T cell differentiation by inhibiting the TLR4/MAPKs/NF-κB axis. These findings also revealed a significant difference in the expression levels of TLR4 between CD4^+^ T cells and B cells. In CD4^+^ T cells, the expression level of TLR4 was relatively low, whereas in B cells, the expression level of TLR4 was relatively high, suggesting that TLR4 may play a more important role in B cells.

## Discussion

In the present study, we provided a comprehensive view of how IRF1 enhances T cell differentiation by recruiting HDAC1 to block B cell CD180 transcription via the TLR4/MAPKs/NF-κB pathway in MG. Our findings indicate that IRF1 plays a crucial role in the pathogenesis of MG by inhibiting the TLR4/MAPKs/NF-κB signaling pathway. This mechanism not only provides a new perspective for understanding the immunopathological process of MG but also offers a theoretical basis for the development of new therapeutic strategies.

IRF1 is crucial for lymphocyte differentiation. In patients with MG, B cells accumulate in the thymus, and MG pathogenesis is linked to disruptions in the balance between T helper 1 and T helper 2 cells (Yi et al., 2018). A study has revealed that mice in which IRF1 regulates the class I auxiliary immune response IRF1^−/−^ are very sensitive to Leishmania major, and its deficiency leads to the transformation of the immune response to quasi-auxiliary humoral immunity (Jayakumar et al., 2008). IRF1 also plays a role in regulating various autoimmune conditions. Leung et al. (2015) reported that IRF1 not only regulates the IFN pathway as a transcription factor, but also participates in the pathogenesis of systemic lupus erythematosus by regulating H4 acetylation. Furthermore, Ren et al. (2011) revealed that IRF1 contributes to the pathogenesis of multiple sclerosis and experimental autoimmune encephalomyelitis. These authors used a cell line with conditional knockout of *IRF1* and overexpressed *IRF1*, observing that this was able to reduce the inflammatory response of autoimmune encephalomyelitis. IRF1 is crucial for the pathogenesis of autoimmune encephalomyelitis by modulating the response of oligodendrocytes to inflammation and injury. However, IRF1 function in MG remains elusive. In the present research, we therefore aimed to further investigate the mechanisms involving IRF1 and CD180 in MG.

As a TLR-like protein, CD180 lacks the intracellular Toll-like IL-1 receptor signaling domain and uniquely forms homodimers, thus playing a role in the development and activation of immune cells only (Schultz and Blumenthal, 2017; Yang et al., 2018). CD180 is linked to the pathophysiology of autoimmune diseases (Edwards et al., 2023). It also mediates activation signals in B cells and is involved in regulating B cell proliferation and apoptosis (Liu et al., 2013). In primary human B cells, CD180 is the primary cell surface marker, and its cross-linking by monoclonal antibodies promotes cell survival and proliferation—an effect that can be reversed by specific inhibitors (Egli et al., 2015). However, the interaction between IRF1 and CD180 has not yet been reported. In the current study, we predicted that IRF1 was a transcription factor bound upstream of CD180 using the JASPAR website, and further confirmed the binding of IRF1 and the *CD180* promoter. Our results revealed that IRF1 inhibited CD180 transcription. The *in vivo* results further demonstrated that IRF1 promoted B cell activation and IRF1 promoted T cell differentiation by inhibiting CD180. Together, our findings suggest that IRF1 may contribute substantially to MG pathogenesis by modulating CD180.

HDAC1 is a unique member of class I HDACs; it is an environmental sensor that regulates endothelial function (Dunaway and Pollock, 2022). The HDAC isomer reportedly regulates inflammation and the autoantibody response in a mouse model of experimental autoimmune MG, and inhibiting HDAC1 reduces IL-6 levels the most. However, HDAC2 inhibition reduces intracellular IL-6 and significantly reduces serum anti-AChR IgG2b in experimental autoimmune MG mice (Bahauddin et al., 2021). In addition, HDAC1 contributes to the activation of CD4^+^ T cells in systemic lupus erythematosus by suppressing miR-124 and enhancing IRF1 expression (Chen et al., 2021). In our study, IRF1 recruited HDAC1 to inhibit CD180 transcription. Moreover, HDAC1 promoted B cell activation and T cell differentiation. Thus, our study revealed the preliminary mechanism of IRF1 with HDAC1 and CD180 in MG.

The pathogenesis of MG is complex and involves the abnormal activation of various immune cells and signaling pathways (Li et al., 2025; Luo et al., 2025). TLRs serve as key detectors in the innate immune system; they identify invariant structural patterns and trigger cellular responses via subsequent signaling cascades (Egli et al., 2015). IRF1 was recently reported to play an important role in regulating immune responses, especially in the activation of B and T cells (Peel et al., 2024). Furthermore, CD180 activation stimulates B cell intrinsic proliferation and differentiation, resulting in a swift rise in IgG levels and the engagement of MyD88-dependent TLR signaling to modulate cell proliferation and differentiation and cytokine secretion (Chaplin et al., 2011). Guo et al. (2016) reported that CD180 protects the myocardium from apoptosis and autophagy and exerts a cardioprotective effect in ischemia/reperfusion injuries, which may be attributable to TLR4/NF-κB pathway inactivation. In addition, in instances of ischemic and septic acute kidney injury, CD180 may provide protection by dampening the TLR4/NF-κB signaling cascade (Zhu et al., 2022). Typically, MG is driven by autoantibodies that target the muscle AChR (Luo and Lindstrom, 2015). AChR is commonly purified from the electric organs of electric fish, which are abundant in this antigen (Fuchs et al., 2014). In AchR^+^ MG, IRF1 regulates MG-specific microRNAs (miR-150-5p, miR-21-5p, and miR-30e-5p) (Fiorillo et al., 2020). The TLR4 activator LPS can effectively replace mycobacterium tuberculosis and induce sensitization to purified AChR (Robinet et al., 2017). Furthermore, our earlier research indicated that a higher count of CD180^−^ B cells in MG is correlated with elevated IgG levels and linked to disease activity and the presence of anti-AChR antibodies (Jin et al., 2021). The present *in vitro* experiments revealed that CD180 suppressed B cell activation and T cell differentiation by impeding the TLR4/MAPKs/NF-κB signaling pathway. Our study therefore further supports the crucial role of IRF1 in the pathogenesis of MG and provides new directions for future research. Additionally, to ensure the specificity and effectiveness of the treatment conditions for the chemical reagents, we conducted dose-dependent experiments, which revealed that 100 ng/mL of LPS and 1 μM of TAK-242 are optimal treatment concentrations. These concentrations not only effectively induced or inhibited the target signaling pathways but also maintained high cell viability, avoiding non-specific effects.

The current study has revealed the important role of IRF1 in regulating CD180 expression; however, there are some limitations. First, our study primarily relied on *in vitro* cell experiments and lacked validation from *in vivo* models. Although our experiments provided important clues for understanding the role of IRF1 in regulating CD180 expression, the absence of an *in vivo* model validation is an important limitation. Future studies should explore the mechanisms of IRF1 in the *in vivo* environment using animal models in order to better reflect the complex immune responses and pathological processes. Second, it is important to note that our study primarily relied on samples from patients with MG, without comparisons to healthy controls or other autoimmune diseases. This limitation means that our findings may not be generalizable to all health and disease states. Future studies should include samples from healthy controls and patients with other autoimmune diseases to more comprehensively evaluate the roles of IRF1 and CD180 in different disease contexts. Additionally, class switching is a key hallmark of B cell activation (Pone et al., 2022), and detecting the relative levels of IgD compared with other Igs (such as IgG, IgA, or IgM) to validate the characteristics of B cell activation may provide more compelling evidence for our study. However, given the time and resource limitations of the current study, we were unable to conduct this experiment. We plan to detect Ig levels in future independent studies to further improve this analysis in our subsequent work. Third, although the current findings indicate that IRF1 suppresses CD180 transcription by recruiting HDAC1, it remains unclear whether other transcription factors or epigenetic regulators might also be involved in this process. For example, might other members of the HDAC family or histone-modifying enzymes also be involved in the regulation of CD180? Future research needs to explore these possibilities to gain a more comprehensive understanding of the regulatory mechanisms of CD180. On the basis of the regulatory mechanisms of IRF1, future research might also explore the possibility of inhibiting inflammatory and autoimmune responses by modulating IRF1 expression or activity. This strategy may offer new therapeutic avenues for MG, especially when existing treatment options are limited in their efficacy. However, the clinical application of this strategy faces many challenges and requires further research to confirm its safety and effectiveness.

In summary, our research explored the mechanisms by which IRF1 is involved in MG. For the first time, we revealed that IRF1 enhanced T cell differentiation via the TLR4/MAPKs/NF-κB pathway in MG by recruiting HDAC1 to block B cell CD180 transcription. Our findings suggest that IRF1 may contribute substantially to MG pathogenesis by modulating CD180. This mechanism not only provides a new perspective for understanding the immunopathological process of MG but also offers a theoretical basis for the development of new therapeutic strategies. Future research should aim to validate these findings in a broader context, including *in vivo* models and comparisons with healthy controls and other autoimmune diseases.

## Additional files:

***Additional Figure 1:*** *Flowchart of the cell experiment.*

Additional Figure 1 Flowchart of the cell experiment.
HDAC1: Histone deacetylase 1; IRF1: interferon regulatory factor 1; LPS: lipopolysaccharide; PMA: phorbol 12-myristate 13-acetate; TAK-242: a Toll-like receptor 4 inhibitor.

***Additional Figure 2:*** *sh-RNA is successfully transfected.*

Additional Figure 2 sh-RNA is successfully transfected.
(A) Assessment of IRF1 expression using RT-qPCR and western blot analysis. (B) Measurement of CD180 levels using RT-qPCR and western blot analysis. *P < 0.05 *vs*. sh-NC. Data are presented as the mean ± SD (*n* = 3) and were analyzed using one-way analysis of variance followed by Tukey's *post hoc* test. IRF1: Interferon regulatory factor 1; RT-qPCR: reverse transcription quantitative polymerase chain reaction.

***Additional Figure 3:*** *Effect of HDAC1 on B cell activation using flow cytometry.*

Additional Figure 3 Effects of HDAC1 on B cell activation using flow cytometry.
(A) Apoptosis rate of B cells. (B) Levels of CD19^+^CD27^+^ in B cells. (C) CD4^+^CD40L^+^ expression in T cells. HDAC1: Histone deacetylase 1.

***Additional Figure 4:*** *Evaluation of HDAC1 expression via RT-qPCR and western blotting.*

Additional Figure 4 Evaluation of HDAC1 expression using RT-qPCR and western blotting.
*P < 0.05 *vs*. sh-NC. Data are presented as the mean ± SD (*n* = 3) and were analyzed using one-way analysis of variance followed by Tukey's *post hoc* test. HDAC1: Histone deacetylase 1; RT-qPCR: reverse transcription quantitative polymerase chain reaction.

***Additional Figure 5:*** *Effects of LPS and TAK-242 on B cells using flow cytometry.*

Additional Figure 5 Effects of LPS and TAK-242 on B cells using flow cytometry.
(A) Apoptosis rate of B cells. (B) CD4^+^CD40L^+^ in T cells. LPS: Lipopolysaccharide; TAK-242: a Toll-like receptor 4 inhibitor.

***Additional Figure 6:*** *Dose-dependent experiments of LPS and TAK-242 on B cells.*

Additional Figure 6 Dose-dependent experiments of LPS and TAK-242 on B cells.
(A) Effects of different concentrations of LPS (0, 25, 50, 100, or 200 ng/mL) on the viability of CD180^−^ B cells treated for 2, 4, and 8 hours. ^$^*P* < 0.05 *vs*. 0 ng/mL LPS. (B) Effects of different concentrations of TAK-242 (0, 0.25, 0.5, 1, or 2 μM) on the viability of CD180^+^ B cells treated for 1 hour. ^H^*P* < 0.05 *vs*. 0 μM. Data are presented as the mean ± SD (*n* = 3) and were analyzed using two-way analysis of variance with the Bonferroni *post hoc* test. LPS: Lipopolysaccharide; OD: optical density; TAK-242: a Toll-like receptor 4 inhibitor.

***Additional Figure 7:*** *Effect of LPS and TAK-242 on sh-CD180 and oe-CD180 cells (flow cytometry).*

Additional Figure 7 Effects of LPS and TAK-242 on sh-CD180 and oe-CD180 cells (flow cytometry).
(A) Apoptosis rate of B cells. (B) CD4^+^CD40L^+^ in T cells. LPS: Lipopolysaccharide; TAK-242: a Toll-like receptor 4 inhibitor.

## Data Availability

*All relevant data are within the paper and its Additional files*.
