# Supplementary figures and images for: Interferon regulatory factor 1 enhances T cell differentiation in patients with myasthenia gravis
Source: Neural Regen Res. 2025 Oct 30;21(7):3267–80. doi: 10.4103/NRR.NRR-D-24-01646 (PMC13379044; doi:10.4103/NRR.NRR-D-24-01646)

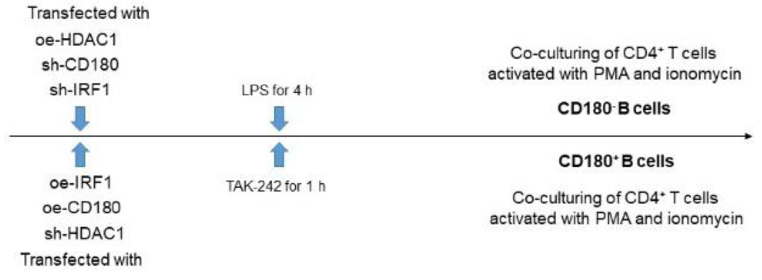

Supplement: Supplementary file 1 [file NRR-21-3267_Suppl1.tif]

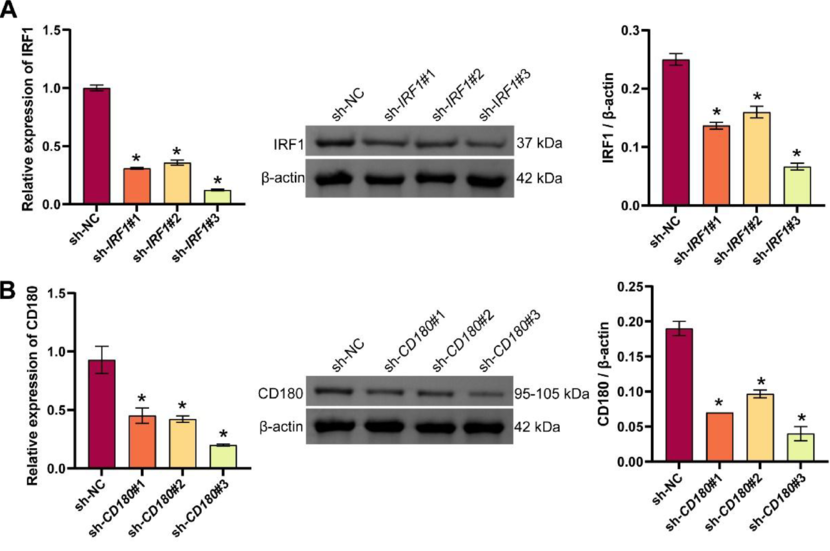

Supplement: Supplementary file 2 [file NRR-21-3267_Suppl2.tif]

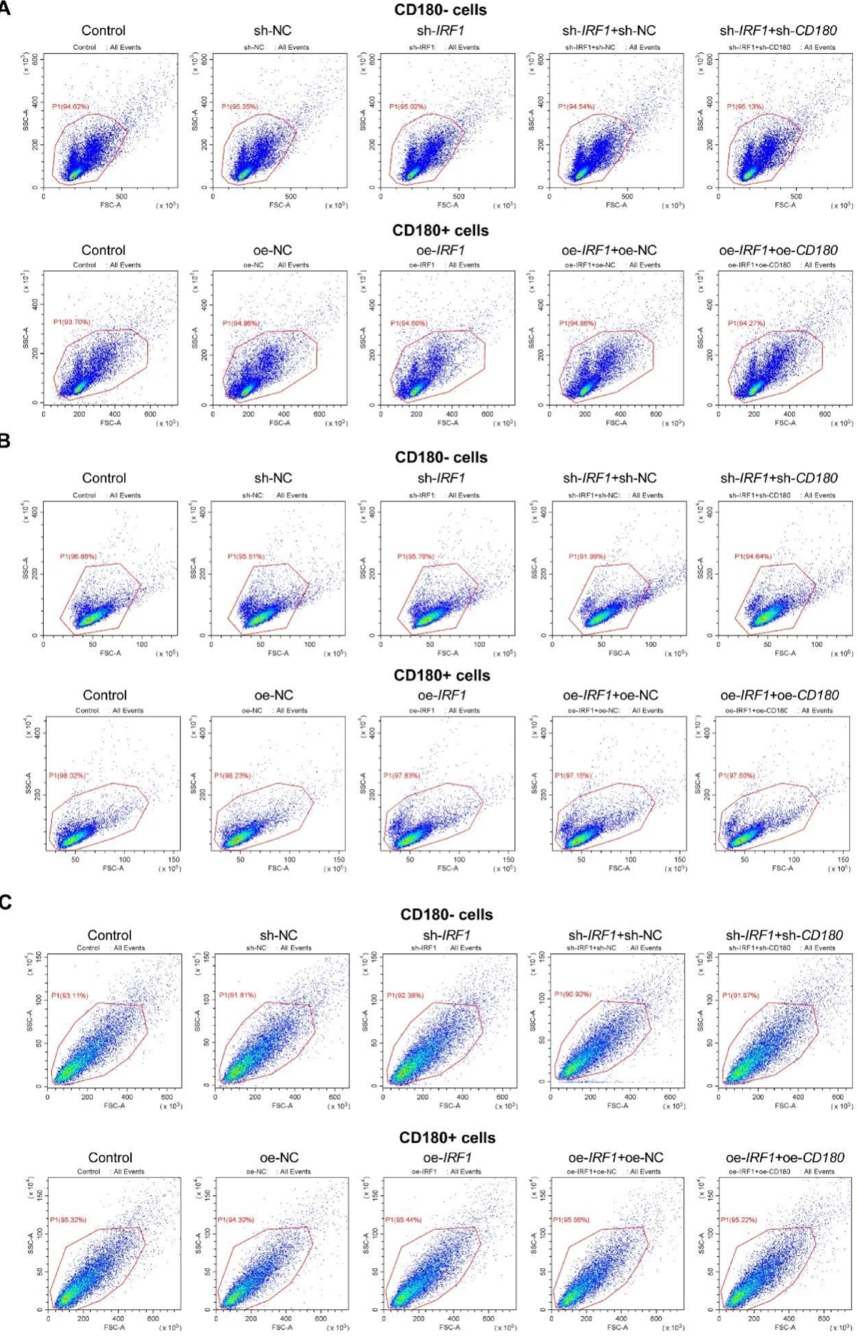

Supplement: Supplementary file 3 [file NRR-21-3267_Suppl3.tif]

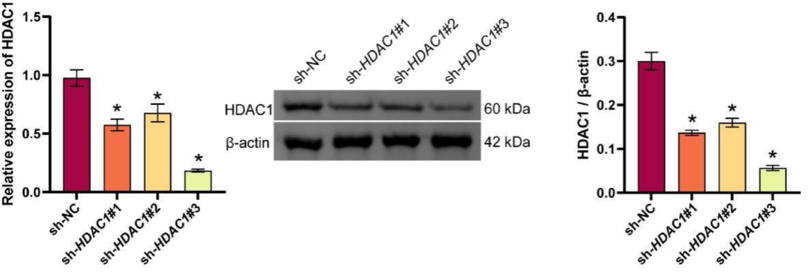

Supplement: Supplementary file 4 [file NRR-21-3267_Suppl4.tif]

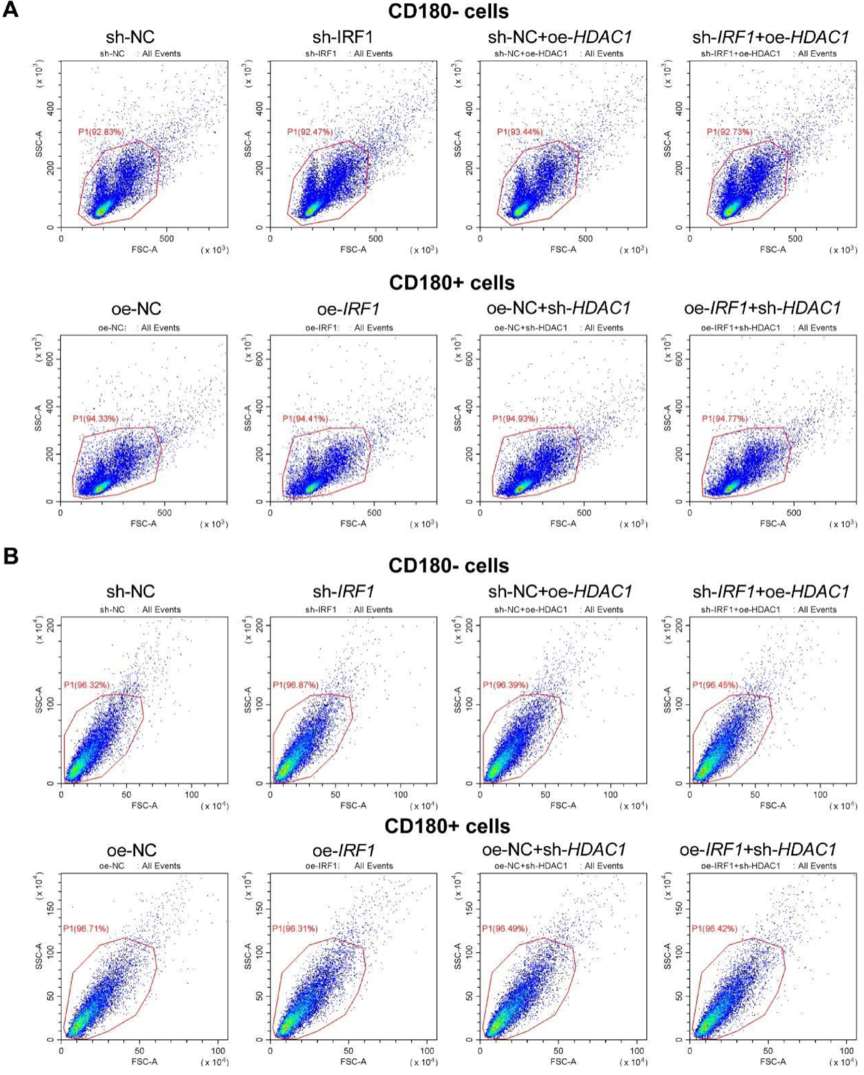

Supplement: Supplementary file 5 [file NRR-21-3267_Suppl5.tif]

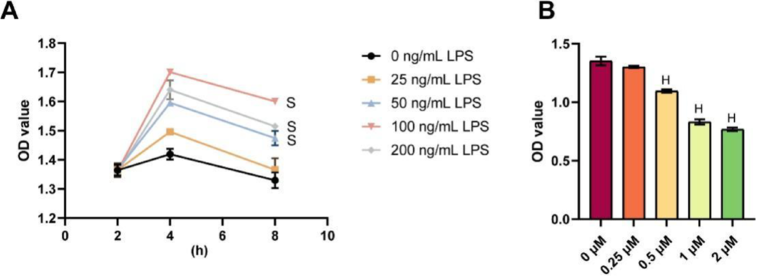

Supplement: Supplementary file 6 [file NRR-21-3267_Suppl6.tif]

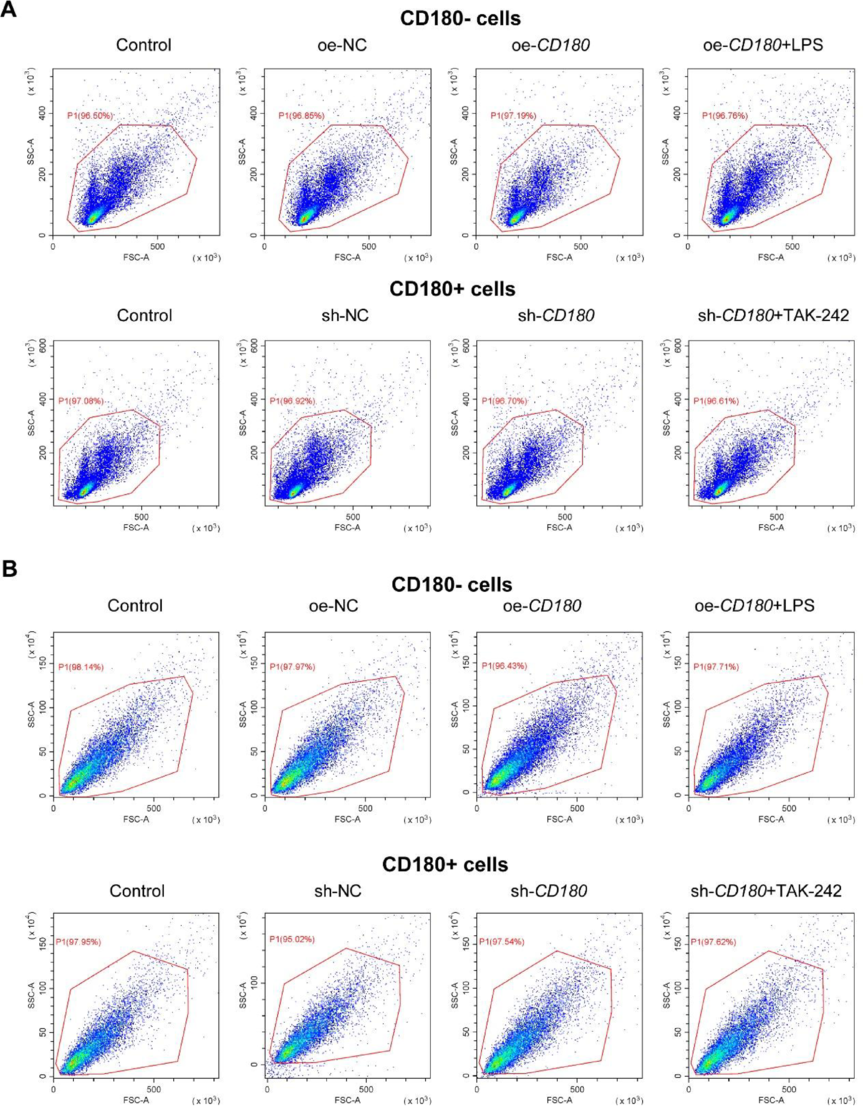

Supplement: Supplementary file 7 [file NRR-21-3267_Suppl7.tif]
